# Prediction of Transposable Element Derived Enhancers Using Chromatin Modification Profiles

**DOI:** 10.1371/journal.pone.0027513

**Published:** 2011-11-07

**Authors:** Ahsan Huda, Eishita Tyagi, Leonardo Mariño-Ramírez, Nathan J. Bowen, Daudi Jjingo, I. King Jordan

**Affiliations:** 1 School of Biology, Georgia Institute of Technology, Atlanta, Georgia, United States of America; 2 National Center for Biotechnology Information, National Library of Medicine, National Institutes of Health, Bethesda, Maryland, United States of America; 3 PanAmerican Bioinformatics Institute, Santa Marta, Magdalena, Colombia; 4 Ovarian Cancer Institute, Georgia Institute of Technology, Atlanta, Georgia, United States of America; National Cancer Institute, United States of America

## Abstract

Experimentally characterized enhancer regions have previously been shown to display specific patterns of enrichment for several different histone modifications. We modelled these enhancer chromatin profiles in the human genome and used them to guide the search for novel enhancers derived from transposable element (TE) sequences. To do this, a computational approach was taken to analyze the genome-wide histone modification landscape characterized by the ENCODE project in two human hematopoietic cell types, GM12878 and K562. We predicted the locations of 2,107 and 1,448 TE-derived enhancers in the GM12878 and K562 cell lines respectively. A vast majority of these putative enhancers are unique to each cell line; only 3.5% of the TE-derived enhancers are shared between the two. We evaluated the functional effect of TE-derived enhancers by associating them with the cell-type specific expression of nearby genes, and found that the number of TE-derived enhancers is strongly positively correlated with the expression of nearby genes in each cell line. Furthermore, genes that are differentially expressed between the two cell lines also possess a divergent number of TE-derived enhancers in their vicinity. As such, genes that are up-regulated in the GM12878 cell line and down-regulated in K562 have significantly more TE-derived enhancers in their vicinity in the GM12878 cell line and vice versa. These data indicate that human TE-derived sequences are likely to be involved in regulating cell-type specific gene expression on a broad scale and suggest that the enhancer activity of TE-derived sequences is mediated by epigenetic regulatory mechanisms.

## Introduction

Transposable elements (TEs) are repetitive genetic sequences that can move from one location in the genome to another. TE-derived sequences are abundant in eukaryotes and make up substantial fractions of their genomic DNA. TEs have long been dismissed as selfish DNA elements that make little or no contribution to the function of their host genomes [1], [2]. This idea was supported by theoretical demonstrations that TEs can persist and proliferate in a genome without providing any function or benefit to the host [3]. In the last couple of decades however, a number of anecdotal cases of TEs contributing regulatory or coding sequences to the host genome were reported. This has led to the development of a more nuanced view of TE sequences, whereby the relationship between TEs and the host genome can be characterized as a continuum ranging from extreme parasitism to obligate mutualism with their host [4], [5]. Indeed, TEs have been implicated in numerous functions that benefit the human genome. One way in which TEs can provide functional utility to the host genome is by donating enhancer sequences that can regulate the expression of host genes.

Enhancers are distal regulatory sequences, found outside of proximal promoter regions, which can increase the expression of genes by interacting with transcription factors. There are a handful of studies that provide experimental evidence for the exaptation of TE sequences as functional enhancers in the human genome. The first example comes from a study in 1993 by Hambor *et al.* which shows that an Alu element serves as part of an enhancer that up-regulates the CD8 alpha gene in accordance with its role in differentiation along the hematopoietic lymphoid lineage [6]. A few years later another study reported that an L1 element sequence donates an enhancer to up-regulate the expression of the APOC (Apolipoprotein) gene by more than 10-fold in cultured hepatocyte cells [7]. Similarly, ancient SINE elements have been shown to serve as enhancers in mammalian specific brain formation. Santangelo et al. demonstrated the selection of a MAR1 element as an enhancer for the POMC (Proopiomelanocortin) gene expressed in the pituitary gland of jawed vertebrates [8]. Another gene FGF8 (fibroblast growth factor 8) has also been shown to be regulated by the AmnSINE1 element in mammalian neuronal tissues [9]. A final study by Bejerano et al. showed that an ancient SINE element drives the expression of ISL1 (insulin gene enhancer protein) in an *in-vivo* mouse enhancer assay [10].

In addition to the experimental evidence showing that individual TE sequences provide functional enhancers to host genomes, we previously found evidence to suggest that human TEs may provide numerous enhancer sequences genome-wide. Our prior analysis showed that TE sequences reside in a substantial fraction of DNaseI hypersensitive (DHS) sites [11]. The location of DHS sites signal ‘open chromatin’ regions which are involved in the regulation of transcription such as promoters and enhancers [12]. The genome-wide analysis of DHS revealed that 23% of these sites contain TE sequences and are associated with higher expression levels of nearby genes in CD4^+^ T-cells [11]. These data suggested that TEs may provide a large number of regulatory sequences that can increase the expression of genes in various tissues. Given the evidence from the experimental cases of TE-derived enhancers and the presence of TE sequences in DHS sites genome-wide, our goal in this study was to further explore the contribution of TEs in donating enhancers to various human cell types.

Experimentally characterized active enhancers display a distinct pattern of chromatin modifications that is significantly different from other regulatory regions as well as the genomic background [13], [14], [15]. Specifically, functionally active enhancers are enriched for a suite of individual histone modifications – H3K4me1, H3K4me2, H3K4me3, H3K9ac, H3K27ac – and their enrichment patterns can be used to predict novel enhancers [13], [14]. We used the chromatin signature of active enhancers to guide the search for putative TE-derived enhancers in two human hematopoietic cell lines, GM12878 and K562, characterized as part of the ENCODE project [16], [17]. We employed a computational approach to identify novel enhancers by building a training set based on ChIP-Seq tag counts of the five enhancer-characteristic histone modifications found over a set of previously defined enhancer regions. Using genome-wide histone modification maps for the GM12878 and K562 cell lines, we identified hundreds of enhancers donated by TEs in each cell line. We also investigated the functional effect of these enhancers on gene expression and observed that TE-derived enhancers play a role in regulating gene expression in a cell type specific manner.

## Results and Discussion

Specific chromatin modification profiles have been shown to mark functionally active enhancer regions in the human genome [13], [14], [15]. We employed a computational approach that uses the patterns of histone modifications to predict novel active enhancers in two human cell lines. The ENCODE project recently characterized genome-wide locations for several histone modifications in different human cell lines [16], [17]. We chose two cell lines derived from the hematopoietic stem cell lineage: GM12878 and K562. GM12878 is a lymphoblastoid cell line derived from a female donor of northern and western European descent, whereas K562 is an immortalized cancer cell line derived from a northern European female patient suffering from immortalized Chronic Myelogenous Leukemia (CML). In each cell line, we analyzed the distribution of eight histone modifications (H3K4me1, H3K4me2, H3K4me3, H3K9ac, H3K27ac, H3K27me3, H3K36me3, H4K20me1) characterized using chromatin immunoprecipitation followed by sequencing (ChIP-Seq). Functional enhancers have also been associated with ‘open chromatin’ as described by DHS sites. Therefore, we also incorporated data of the genomic locations of DHS characterized by the ENCODE project in GM12878 and K562 cells [16], [17].

### Enhancer training set

**Figure 1:**
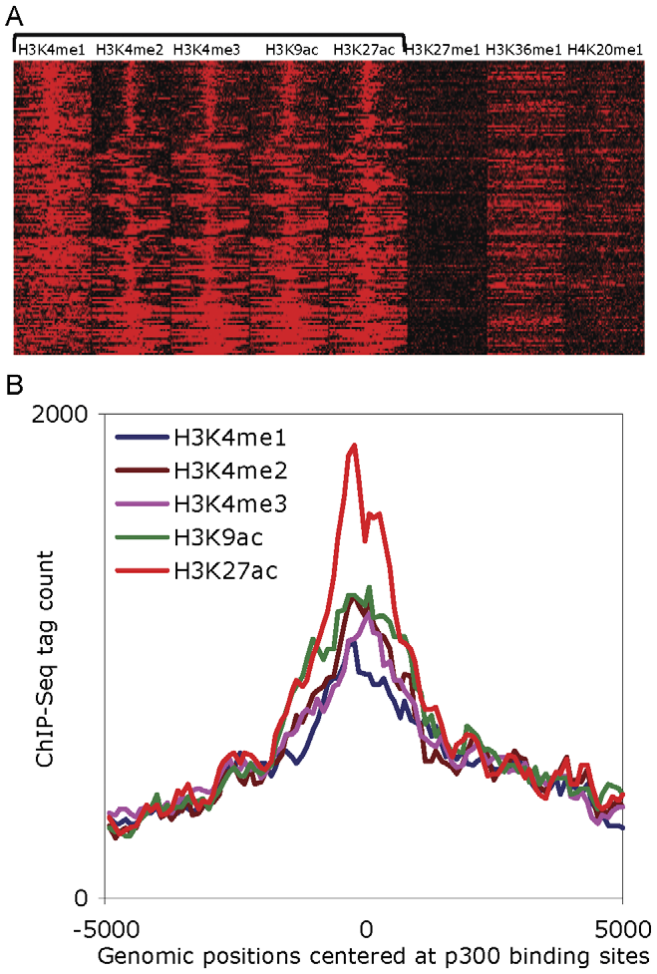
An enhancer training set based on histone modification enrichment. The enhancer training set is derived from five histone modifications in 10 KB windows over 137 p300 binding sites in the K562 cell line. (A) Heat map showing ChIP-Seq tag counts at 137 p300 binding sites for eight histone modifications. The first five of the modifications are significantly enriched and display distinctive patterns at p300 binding sites, whereas the last three modifications do not show any specific pattern over p300 binding sites. (B) Visual representation of the enhancer training set with ChIP-seq tag counts summed over 137 p300 bound genomic loci corresponding to the five enhancer enriched histone modifications binned in 100 bp bins over a 10 kb window.

Functionally active enhancers are marked by an enrichment of the transcription co-activator protein p300 [18], [19]. As an integral part of the enhancer-associated protein complex, p300 has been found at enhancer locations across the human genome [20], [21], [22]. A set of p300 bound genomic locations has recently been characterized using the ChIP-chip technique in human K562 cells, and these p300 binding sites have been taken to represent a genome-wide map of functional enhancers [13], [14]. In order to determine the chromatin modification profile of enhancers, we evaluated eight histone modifications at experimentally characterized p300 binding sites in the K562 cell line. We found that five of these modifications (H3K4me1, H3K4me2, H3K4me3, H3K9ac, H3K27ac) display distinct patterns at p300 binding sites that can be used to predict putative enhancers (Figure 1). Therefore, we selected 137 of the p300 binding sites that are significantly enriched for all five modifications above the genomic background to build an enhancer training set. The training set consists of five vectors, each representing ChIP-Seq tag counts of individual histone modifications over a 10 kb region divided in 100 bp bins and summed over the 137 p300 binding sites (see Methods).

We employed two controls to validate the discriminatory power of the chromatin modification profile captured by our enhancer training set. As a first control, we compared the genomic location profiles for mapped ChiP-Seq tags of the five enhancer enriched histone modifications with the three remaining modifications around the 137 p300 binding sites. The five enhancer enriched histone modifications present in our training set (H3K4me1, H3K4me2, H3K4me3, H3K9ac, H3K27ac, H3K27me3) display unique patterns of enrichment, with tag count peaks centered around the p300 binding sites, whereas the other three histone modifications (H3K27me3, H3K36me3, H4K20me1) do not show any specific pattern of enrichment over enhancer regions (Figure S1). As a second control, we sampled 137 random genomic sequences and compared the profiles of histone modification tag counts against those in our training set derived from p300 binding sites. We observed that random genomic locations do not display any pattern of histone modification enrichment characteristic of experimentally characterized enhancers (Figure S2). Taken together, these controls demonstrate that experimentally characterized enhancers display unique patterns of enrichment of five histone modifications, which are significantly different from the genomic background. Thus, the epigenetic histone modification profile captured by our enhancer training set possesses the discriminating features necessary to search for novel enhancers.

We attempted to further ascertain the discriminating power of our enhancer training set by performing cross-validation together with receiver operating characteristic (ROC) analysis on the genomic loci that constitute our enhancer training set (see Methods). The resulting ROC curve provides a graphical method to distinguish between optimal and suboptimal models in their diagnostic ability. The curve is plotted as the rate of true positives against false positives at given intervals, and the departure of the optimal model from the unity line is taken as a measure of its performance.

**Figure 2:**
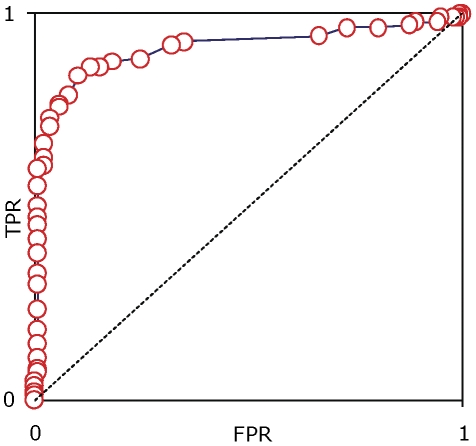
Discriminating ability of the enhancer training set. Receiver operating characteristics (ROC) curve showing the discriminating ability of the enhancer training set. The rate of true positives (TPR) is calculated as the correlations between the chromatin profiles of individual sequences that make up the enhancer training set with the entire enhancer training set considered as an ensemble, and the rate of false positives is calculated as the correlations between the chromatin profiles of randomly sampled genomic loci and the training set (see Methods). Departure of the curve from the unity line is taken as a measure of the discriminating ability of the training set.

To plot the ROC curve, for each of 10 cross-fold validations, we computed Spearman's rank correlations (ρ) between ChIP-seg tag counts that represent the chromatin profile of the enhancer training sets as an ensemble against tag counts for the p300 bound regions that make up the validation sets (true positives). Then, as a control, we performed similar correlations between the chromatin profile of the training sets and a sets of random genomic loci the same size of the validation sets (false negatives). Across a range of ρ-values, the fraction of true positives represented by the validation set loci was plotted against the fraction false positives from random genomic loci to yield the ROC curve in Figure 2. The plot demonstrates that our enhancer training set is clearly distinct from the one derived from sampling random genomic loci and thereby possesses the discriminating capability essential for its use in enhancer predictions.

### Enhancer prediction

Having established the validity of our enhancer training set, we used it to search for regions that display similar chromatin profiles in order to identify potential TE-derived enhancers, which may or may not be bound by p300, genome-wide. To that end, we built a test set made up of the DHS sites in the GM12878 and K562 cell lines. Using a 10 kb window and a step size of 100 bp, we computed Spearman's rank correlations between the enhancer training set histone modification profile and test set profiles at each step. For each DHS site, the genomic site that yields the highest correlation value was recorded, and the results were filtered using a correlation cut-off of 0.5 or higher (Spearman's ρ = 0.5, *n* = 98, *P* = 1E−7).

**Figure 3:**
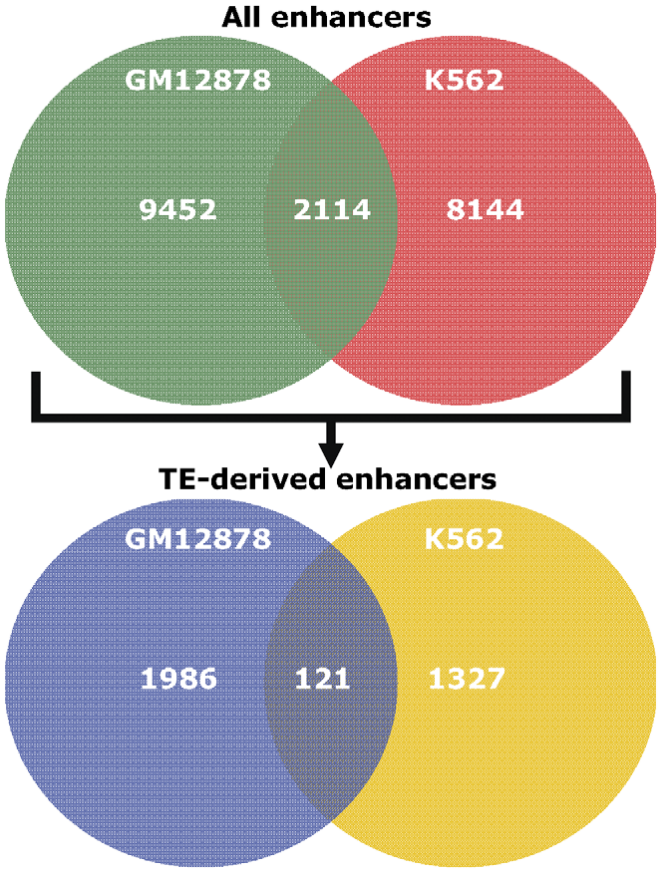
Common and exclusive enhancers between the GM12878 and K562 cell lines. (Top) Venn diagram showing the numbers of enhancers that are shared between the GM12878 and K562 cell lines as well as unique enhancers in the two cell lines, and (bottom) numbers of the enhancers from above that originate in TE sequences are shown.

Several histone modifications are also known to be enriched at the transcription start sites and promoter regions of human genes [13], [15]. Since actively transcribing genes are also associated with DHS, there is a possibility that our enhancer prediction method can potentially misidentify some promoters as enhancers. In order to control for this possibility, we used CAGE (Cap Analysis of Gene Expression) data in each cell line to filter any promoters that may have been identified as enhancers. CAGE tags are obtained by capping the 5′ end of messenger RNA and are known to mark the transcriptional start sites of genes [23], [24]. We identified loci that are significantly enriched for CAGE tags by using a Poisson distribution parameterized by the background CAGE tag count [16], [17]. The potential enhancer predictions that overlapped with CAGE tags were marked as promoters and removed from consideration. After filtering out the promoters in this way, (Delete – CAGE analysis removed) we obtained 11,311 and 8,051 enhancers in the GM12878 and K562 cell lines respectively. A majority of enhancers we identified are unique to each cell line as only 2,114 (10.7%) of these enhancers are shared between both (Figure 3).

Since we limited our search for enhancers to DHS sites, these data reflect the number of enhancers associated with actively transcribing genes. Histone modifications in each cell type are dynamic and can change to accommodate the regulatory needs of cell. Thus, the enhancers predicted using the histone modification profiles are also not universally active as reflected by the small percentage of enhancers that are shared between GM12878 and K562 cell lines. As such, these figures provide a snapshot of active enhancers in two human cell lines, each going through a particular stage of differentiation. Accordingly, the divergent genomic loci of these enhancers suggest their role in regulating cell type specific gene expression as discussed in later sections.

### TE-derived enhancers

In order to identify functionally active TE-derived enhancers, we intersected the genomic loci of our predicted enhancers in each cell line with TE annotations produced by the RepeatMasker program [25]. We identified 2,107 and 1,448 enhancers derived from TEs in the GM12878 (Table S1) and K562 (Table S2) cell lines respectively with 121 (3.5%) enhancers that are shared between both cell lines (Figure 3 and Table S3). There is a significantly smaller fraction of TE-derived enhancers that are common between the cell lines compared to all predicted enhancers (Hyper-geometric test, *P* = 2E−63), suggesting that TE-derived enhancers are more cell type specific.

**Figure 4:**
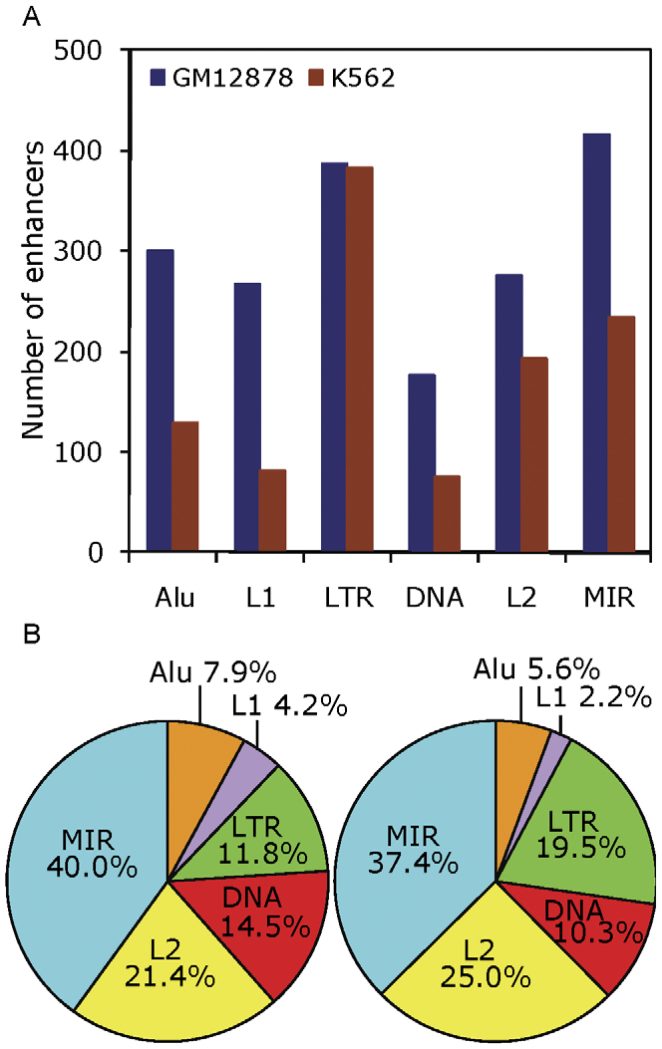
Contribution of various TE families in providing enhancers to the human genome. (A) The number of enhancers provided by six TE families in GM12878 (blue) and K562 (brown) cell lines. (B) Contribution of enhancers by TE families normalized by their genomic abundance (see Methods).

To evaluate the contribution of various families of TEs in donating enhancers, we divided TE-derived enhancers into 6 major families, based on the Repbase classification system [26], [27], namely Alu, L1, LTR, DNA, L2, and MIR (Figure 4A). We also normalized the number of enhancers contributed by each TE family by the family's relative genomic abundance (Figure 4B). In both cell lines, Alu and L1 elements are under-represented, whereas LTR, DNA, L2 and MIR are over-represented TE families that contribute enhancers to the human genome (χ^2^ test, GM12878: *P* = 9E−272, K562: P = 4E−217 –Table S4, Student's *t* test, GM12878: *t* = 5.6, *P* = 1E−3, K562: *t*  = 4.6, *P* = 3E−3). In absolute terms, LTR elements donate the highest number of enhancers (387) in the K562 and the second highest number of enhancers (383) in the GM12878 cell lines. A number of previous studies have demonstrated that LTRs provide transcription start sites and protein coding sequences to the human genome [28], [29]. Thus, our analysis extends what is known regarding the extensive regulatory contributions of LTR elements to the human genome. Our data also indicate that MIR elements contribute the largest number of enhancers relative to their genomic abundance. MIRs represent the oldest family of TEs in the human genome, and their over-representation in donating enhancers indicates that older TEs are more likely to provide regulatory and coding sequences for the host genome [30]. Indeed, the relative age of TE families is directly correlated with the number of enhancers it donates (Figure S3, GM12878: ρ = 0.94, *P* = 3E−19, K562: ρ = 0.89, *P* = 2E−17). The observation that older TE families donate relatively more enhancers than younger ones suggests that older elements may possess a stronger ability to recruit epigenetic marks, making them more likely to be exapted by the host genome. We have also previously shown that older TEs are bear more histone modifications than younger ones and therefore demonstrate a higher potential to be exapted by the human genome [31].

### TE-derived enhancers and cell type specific gene expression

To evaluate the functional effect of TE-derived enhancers, we investigated their role in the regulation of gene expression. Enhancers can influence the expression of genes that lie as many as tens-of-thousands of bases away from the transcriptional start site of genes. We determined the functional effect of our predicted TE-derived enhancers by relating them to cell type specific gene expression. To do this, we mapped enhancers to genes by finding enhancers in 100 kb windows surrounding transcriptional start sites.

**Figure 5:**
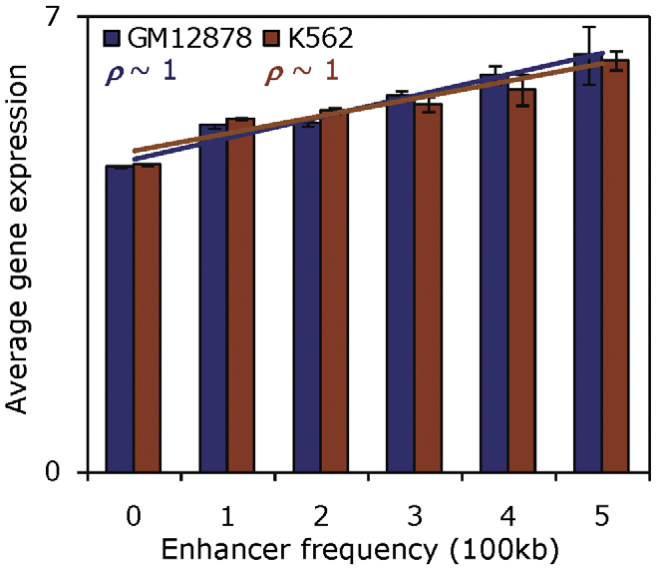
Functional role of TE-derived enhancers in regulating gene expression. Average expression levels (y-axis) of genes that are co-located with different numbers of TE-derived enhancers (x-axis) shown for GM12878 (blue) and K562 (brown) cell lines.

We analyzed GM12878 and K562 gene expression data characterized by exon array experiments as part of the ENCODE project (see Methods) and calculated the average expression of genes that possess different numbers of TE-derived enhancers in their vicinity. For each gene in our dataset, we searched a window of 100 kb surrounding its transcription start site for TE-derived enhancers and binned the average expression of genes with respect to the number of enhancers they possess. The expression of genes without a TE-derived enhancer is significantly lower than that of genes with one or more TE-derived enhancers in the 100 kb region surrounding their transcription start sites (Students' *t* test, GM12878: *t* = 31.2, *P* = 3E−208; K562: *t* = 31.4, *P* = 4E−211). Furthermore, the expression level of genes is strongly positively correlated with the number of TE-derived enhancers it has in its vicinity (Figure 5) (Spearman's GM12878: ρ∼1, *p* = 3E−3, K562: ρ∼1, *p* = 3E−3). These findings suggest that TE-derived enhancers make a contribution to the up-regulation of the expression of nearby genes. As a control, we did the same analysis using non-TE derived enhancer sequences predicted in the same way; the results are qualitatively identical underscoring the potential functional significance of TE-derived enhancers (Figure S4).

### TE-derived enhancers and differential expression

**Figure 6:**
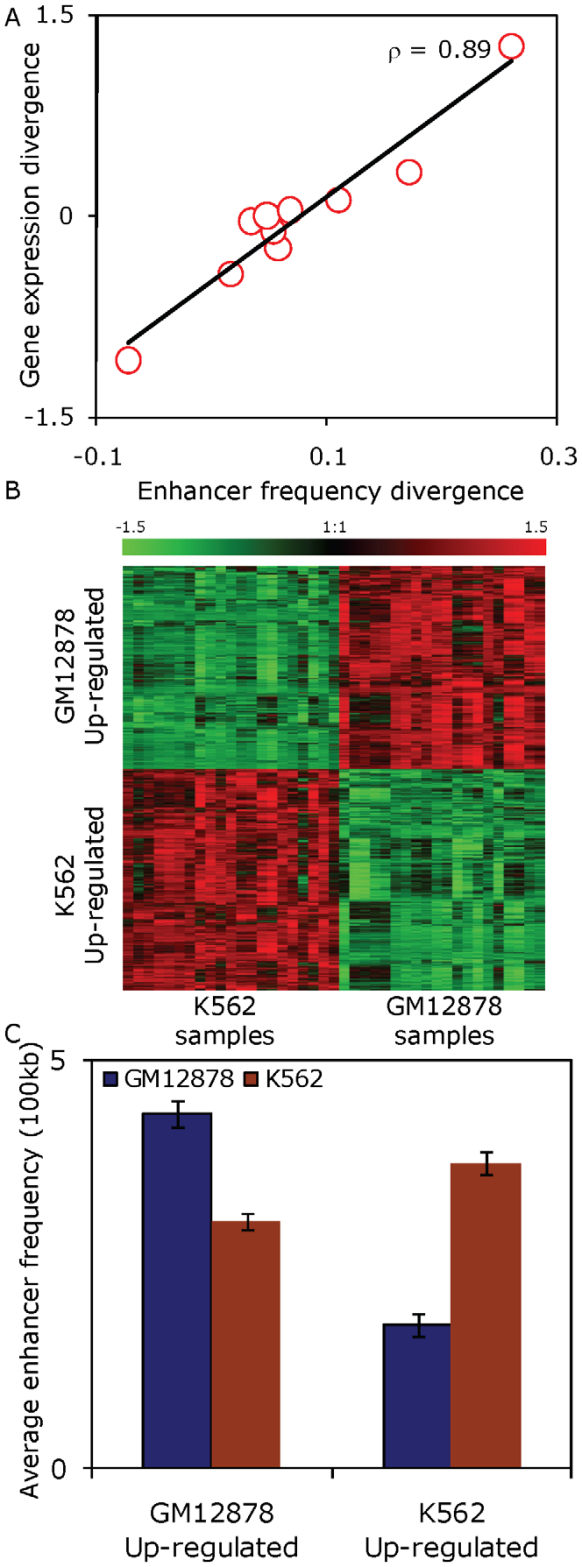
Functional role of TE-derived enhancers in regulating differential gene expression. (A) Gene expression divergence between GM212878 and K562 (y-axis) is plotted against normalized differenced in the numbers of cell type specific TE-derived enhancers (x-axis) co-located with the genes. Expression divergence and enhancer frequency divergence between the GM12878 and K562 cell lines is calculated by subtracting the values of K562 from those of GM12878 cell line. (B) Differentially expressed genes determined by performing ANOVA on the 21 and 20 samples of GM12878 and K562 cell lines respectively (see Methods). (C) The average numbers of co-located TE-derived enhancers found in the GM12878 (blue) and K562 (brown) cell lines are shown for differentially expressed genes that are up-regulated in GM12878 and K562.

Having established the likely functional relevance of TE-derived enhancers in regulating cell type specific gene expression, we evaluated their role in driving differential expression between cell lines. For each gene in our dataset, we computed expression divergence between the GM12878 and K562 cell lines and related it to the difference in the number of gene associated TE-derived enhancers between the two cell lines. We sorted the genes based on their expression divergence and binned them into ten bins according to their increasing expression divergence. We found that expression divergence is directly correlated with the difference in the number of TE-derived enhancers between the GM12878 and K562 cell lines (Figure 6; Spearman's ρ = 0.89, *P* = 1E−3). In addition, the trend is not entirely linear; the most extreme bins on either end show the greatest relationship between gene expression divergence and TE-enhancer frequency divergence. This suggests that the strongest influence of TE-derived enhancers is observed for the most differentially expressed genes. Non-TE derived enhancers show a similar, if more linear and stronger, positive correlation between gene expression divergence and enhancer divergence (Figure S5).

In order to further investigate this phenomena, we used ANOVA on 20 and 21 samples of normalized exon array data from the GM12878 and K562 cell lines respectively to determine the maximally differentially expressed genes. We found 4,118 genes that are significantly differentially expressed (*P* = 1E−7) with 1,970 genes that are up-regulated in GM12878 and down-regulated in K562 and 2,148 genes that are down-regulated in GM12878 and up-regulated in K562 cell lines (see Methods). We computed the average number of enhancers in a 100 kb window surrounding differentially expressed genes and found that genes that are up-regulated in one cell line have more enhancers in their vicinity in the same cell line compared to the other cell line (Figure 6). In our dataset of 1,970 genes that are up-regulated in GM12878 and down-regulated in K562, there are an average of 0.43 TE-derived enhancers per gene in GM12878 and 0.17 TE-derived enhancers per gene in K562 cell line (Wilcoxon signed-rank test, *W* = 183,472, *P* = 1E−37). Similarly, the 2,148 genes that are up-regulated in K562 and down-regulated in GM12878 have 0.37 TE-derived enhancers per gene in K562 and 0.30 TE-derived enhancers per gene in GM12878 cell line (Wilcoxon signed-rank test, *W* = 143,897, *P = 4E*−*4)*. These analyses demonstrates that there are more TE-derived enhancers present near genes that are differentially up-regulated in one cell line versus the other, highlighting their contribution to the regulation of differential expression between cell types.

Since we are comparing two cell lines here, it is formally possible that the differences in expression between cell lines are not related to up-regulation of genes associated with cell-type specific TE-derived enhancers in one cell line. Rather, it may be that the corresponding enhancer sequences, which bear distinct chromatin profiles in the alternate cell line, are actually exerting some negative regulatory effect therein. To control for this possibility, we compared the levels of expression for genes associated with cell-type specific TE-derived enhancers against the expression levels of all genes within cell types. We found that the genes associated with cell-type specific TE-derived enhancers are expressed at significantly higher levels than other genes within the same cell type (Student's ttest; GM12878, t = 18.9, *P* = 2E−73; K562, t = 40.0, *P* = 4E−216). These results are consistent with a positive regulatory role, *i.e.* activation of expression, for the cell-type specific TE-derived enhancers identified here.

### Conclusions

Unlike promoters, enhancers can influence the expression of genes that lay tens-of-thousands of bases away from them [32], [33]. The distribution of TE derived enhancers around genes ranges from hundreds of bases from the transcription start site to several thousand bases. Enhancers in general have also been shown to provide for the most cell type specific mode of gene regulation [13], and the locations of the TE-derived enhancers we discovered here are even more cell type specific than those of the non-TE-derived enhancers. We used two metrics to investigate the possible functional role of TE-derived enhancers in regulating the expression of genes in a cell type specific manner. Our first analysis revealed that the frequency of TE-derived enhancers in the vicinity of genes is strongly correlated with increasing gene expression. Secondly, genes that are differentially up-regulated in each cell line possess significantly more TE-derived enhancers in the same cell line when compared to the other cell line. These results provide evidence for the functional relevance of TE-derived enhancers in helping to differentially regulate genes between human cell types. Nevertheless, experimental interrogation of individual TE-derived enhancers sequences predicted here will be needed to validate the extent and nature of their regulatory activity. We hope that the list of predicted TE-derived enhancers that results from this work can serve as a guide for further experimental studies on the regulatory contributions of human TEs.

## Methods

### Enhancer training set and identification of novel enhancers

A dataset of 211 p300 binding sites characterized genome-wide from the K562 cell line was taken to represent functionally active enhancer sequences as previously described [13], [14]. We downloaded genome-wide ENCODE histone modification ChiP-Seq data [16], [17] for the GM12878 and K562 cell lines from the USCS Genome Browser for 8 histone modifications characterized in the Bernstein Laboratory at the Broad Institute [34], [35], [36]: H3K4me1, H3K4me2, H3K4me3, H3K9ac, H3K27ac, H3K27me1, H3K36me1, H4K20me1. ChIP-Seq tags were mapped to the human genome (UCSC hg18) using the program MAQ with the read-rescue option, which accommodates ambiguous tags that map to multiple genomic regions.

Previously, enhancer locations were predicted using chromatin profiles based on three histone modifications [13]. Here, we have taken a similar approach using additional information afforded by a total of five enhancer-characteristic modifications as well as DHS sites. DHS sites analyzed were aggregated across 10 kb windows and represent relatively open chromatin; although, they are not entirely devoid of nucleosomes. The p300 binding sites were evaluated for enrichment with five enhancer-characteristic histone modifications: H3K4me1, H3K4me2, H3K4me3, H3K9ac, H3K27ac. The remaining three histone modifications (H3K27me1, H3K36me1, H4K20me1) were used as negative controls for enhancer regions. A total of 137 p300 binding sites in K562 cells that were found to be significantly enriched for all five enhancer-characteristic histone modifications, but not for enhancer-negative modifications, were used to predict the locations of TE-derived enhancers genome-wide.

For each histone modification, enrichment significance was calculated using a Poisson distribution parameterized by the genomic background ChIP-Seq tag count and the threshold was adjusted using the Bonferroni correction for multiple tests. The enhancer training set was generated using 10 kb windows center-aligned and surrounding 137 p300 binding sites and divided into 100 bins of 100 bp each. Thus, the training set consists of five vectors representing individual histone modifications each made up of 100 bins containing ChIP-Seq tag counts summed over 137 p300 binding sites.

The test set vectors were fashioned in a similar way except in this case individual DHS sites were used instead of p300 binding sites as in the training set. Individual enhancer test set profiles were centered at the start point of the DHS sites and Spearman's rank correlations were computed individually between the five vectors of the training and the test set profiles and the resulting correlations were averaged. We used a sliding window with a step of 100 bp from the start of the DHS sites, computed correlations at every step and took the highest average correlation computed from all the steps within a DHS site. Average Spearman's correlation values of (ρ = 0.5, *P* = 1E−7) or higher were taken for further evaluation as potential enhancers.

### Cross-validation and receiver operating characteristic (ROC) curve analysis

Ten-fold cross-validation was combined with ROC analysis to evaluate the discriminating power of the enhancer histone modification model. For the 10-fold cross-validation, we partitioned the training set of 137 p300 binding sites into 10 subsamples (with 13 or 14 sequences each), taking 9 of the subsamples as the histone modification training set and a single subsample as the validation set for testing the model. This procedure was iterated 10 times with each of the subsamples used one time as the validation set for testing the model. Then for each cross-validation, Spearman rank correlation coefficients (SCCs) between the histone modification profile of the enhancer training set versus the histone modification profiles of the validation set were computed along with SCCs for the histone modification profile of the training set versus modification profiles of a set of random genomic sequence the same size as the validation set. The resulting ROC curve is based on the relative distributions of the SCC for all ten of the enhancer training versus validation sets (true positives) along with the SCC all ten of the enhancer training versus random genomic sequence sets (false positives). The rates of true positives and false positives were calculated by taking the normalized frequency of correlation values *i* at regular intervals as described below:
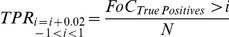


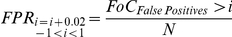



where interval *i* = 0.02, *N* = 137 (total number of correlation values in each dataset), and *FoC* = frequency of correlation values in range. The rate of true positives (TRP) was plotted against the rate of false positives (FPR) to yield the ROC curve in Figure 2.

### Gene expression analysis

We downloaded Affymetrix exon array signal intensity data from the GEO database under accession number GSE12760. This dataset contains 20 samples of GM12878 and 21 samples of K562 cell lines collected from the different laboratories that are part of the ENCODE project [16], [17]. We normalized the dataset using the MAS5 algorithm provided by the Bioconductor package Exonmap [37]. The normalized data was mapped to a genomic locus by averaging the expression values of all probes whose genomic coordinates lay within that the boundaries of that locus for all replicates. We used Refseq genes from the UCSC genome browser to define transcriptional units (TU) [15], [38], [39]. The TU's, referred to as genes in the text for clarity, encompass the all overlapping co-directional mRNA transcripts at a genomic loci. We defined the boundaries of TUs as the upstream most transcription start site and the downstream most transcription end site.

### Differentially expressed genes

Differentially expressed genes were identified using one way ANOVA (Analysis of variance) implemented in the Genesis software package [40]. ANOVA was performed on 20 and 21 samples from GM12878 and K562 cell lines respectively. We used a stringent significance cut-off of *P* = 1E−7 obtained after using Bonferroni correction for multiple tests, to calculate ANOVA.

### Sequence annotation datasets

We used five sequence annotation datasets from the March 2006 build (NCBI Build 36.1; UCSC hg18) of the human genome. Three of these datasets were obtained from the ENCODE section of the UCSC Genome Browser [38]. These datasets include histone modifications, DNaseI hypersensitive sites and CAGE data, for both GM12878 and K562 cell lines [16], [17]. These data are produced from ChIP-Seq, DNaseI-Seq and CAGE experiments respectively and are available as aligned reads in tagAlign files. Refseq genes and RepeatMasker 3.2.7 data was downloaded from the UCSC Table Browser [39].

### Statistical analyses

We used the statistical software R for calculating the Spearman's rank correlation coefficients ρ for all correlation analyses. The statistical significance of Spearman's rank correlation coefficients ρ was determined using the Student's *t* distribution with *d.f.* = n−2 with the formula 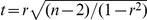 [41].

We used a two tailed χ^2^ test with *d.f.* = 5 and Student's *t* test with *d.f.* = 1 to determine the statistical significance of the over- and under-represented TE-families that donate predicted enhancers in each cell line (Figure 4 and Table S4). The genomic abundance of TE families was used to compute the expected number of enhancers derived from each family.

The Wilcoxon signed-rank test was used to establish the statistical significance for the difference in the average number of enhancers in the vicinity of genes that are differentially expressed between each cell line (Figure 6C).

## Supporting Information

Figure S1 **Control 1: Relevant versus non-relevant histone modifications.** Histone modifications at 137 p300 binding sites in the K562 cell line are shown. The first five modifications were used to build the training set (H3K4me1, H4K4me2, H3K4me3, H3K9ac, H3K27ac), whereas other modification that show no specific pattern of enrichment over the p300 binding sites and were thus excluded from further analysis (H3K9me1, H3K27me3, H3K36me3, H4K20me1).
(PPT)

Figure S2 **Control 2: Histone modification enrichment patterns at p300 binding sites versus random genomic loci.** Epigenetic histone modification levels at 137 p300 binding sites as well as 137 random genomic loci in the K562 cell line are shown. Random genomic loci do not show any discernable pattern of histone modification enrichment compared to p300 binding sites.
(PPT)

Figure S3 **Over and under-represented TE families in contributing enhancers.** Number of TE-derived enhancers observed for different TE families in the GM12878 (blue) and K562 (brown) cell lines normalized by the relative genomic abundances of TE families.
(PPT)

Figure S4 **Functional role of non TE-derived enhancers in regulating gene expression.** Average expression levels (y-axis) of genes that are co-located with different numbers of non TE-derived enhancers (x-axis) shown for GM12878 (blue) and K562 (brown) cell lines.
(PPT)

Figure S5 **Functional role of non TE-derived enhancers in regulating differential gene expression.** Gene expression divergence between GM212878 and K562 (y-axis) is plotted against normalized differenced in the numbers of cell type specific non TE-derived enhancers (x-axis) co-located with the genes. Expression divergence and enhancer frequency divergence between the GM12878 and K562 cell lines is calculated by subtracting the values of K562 from those of GM12878 cell line.
(PPT)

Table S1 **1,986 TE-derived enhancers in the GM12878 cell line.**
(TXT)

Table S2 **1,127 TE-derived enhancers in the K562 cell line.**
(TXT)

Table S3 **121 TE-derived enhancers shared between the GM12878 and K562 cell lines.**
(TXT)

Table S4 **χ^2^ statistics for over and under represented TE families in contributing enhancers.**
(PPTX)
