# Supplementary figures and images for: Prediction of Transposable Element Derived Enhancers Using Chromatin Modification Profiles
Source: PLoS One. 2011 Nov 7;6(11):e27513. doi: 10.1371/journal.pone.0027513 (PMC3210180; doi:10.1371/journal.pone.0027513)

## Slide 1
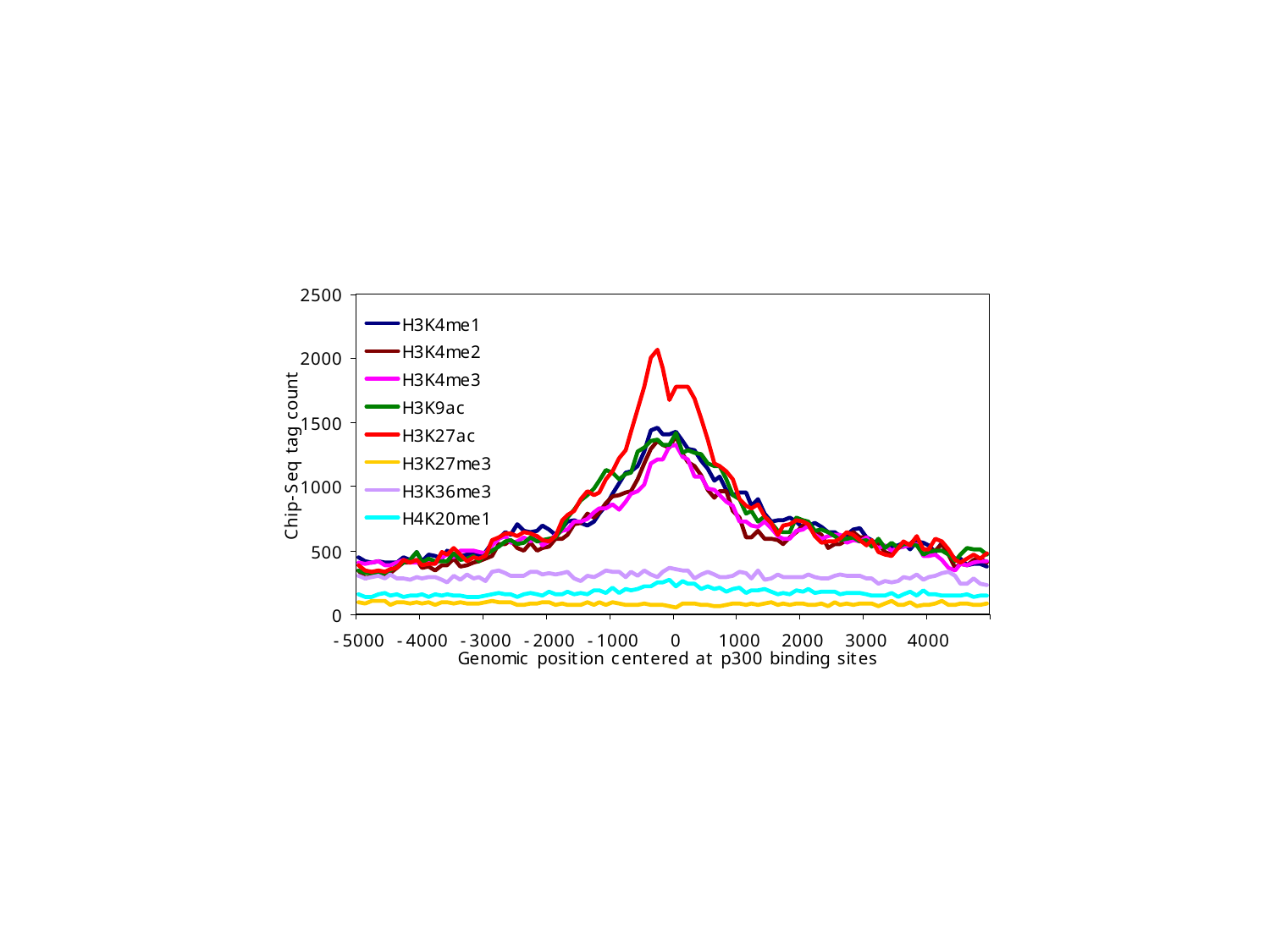

Supplement: Figure S1 — Control 1: Relevant versus non-relevant histone modifications. Histone modifications at 137 p300 binding sites in the K562 cell line are shown. The first five modifications were used to build the training set (H3K4me1, H4K4me2, H3K4me3, H3K9ac, H3K27ac), whereas other modification that show no specific pattern of enrichment over the p300 binding sites and were thus excluded from further analysis (H3K9me1, H3K27me3, H3K36me3, H4K20me1). (PPT) [file pone.0027513.s001.ppt]

## Slide 1
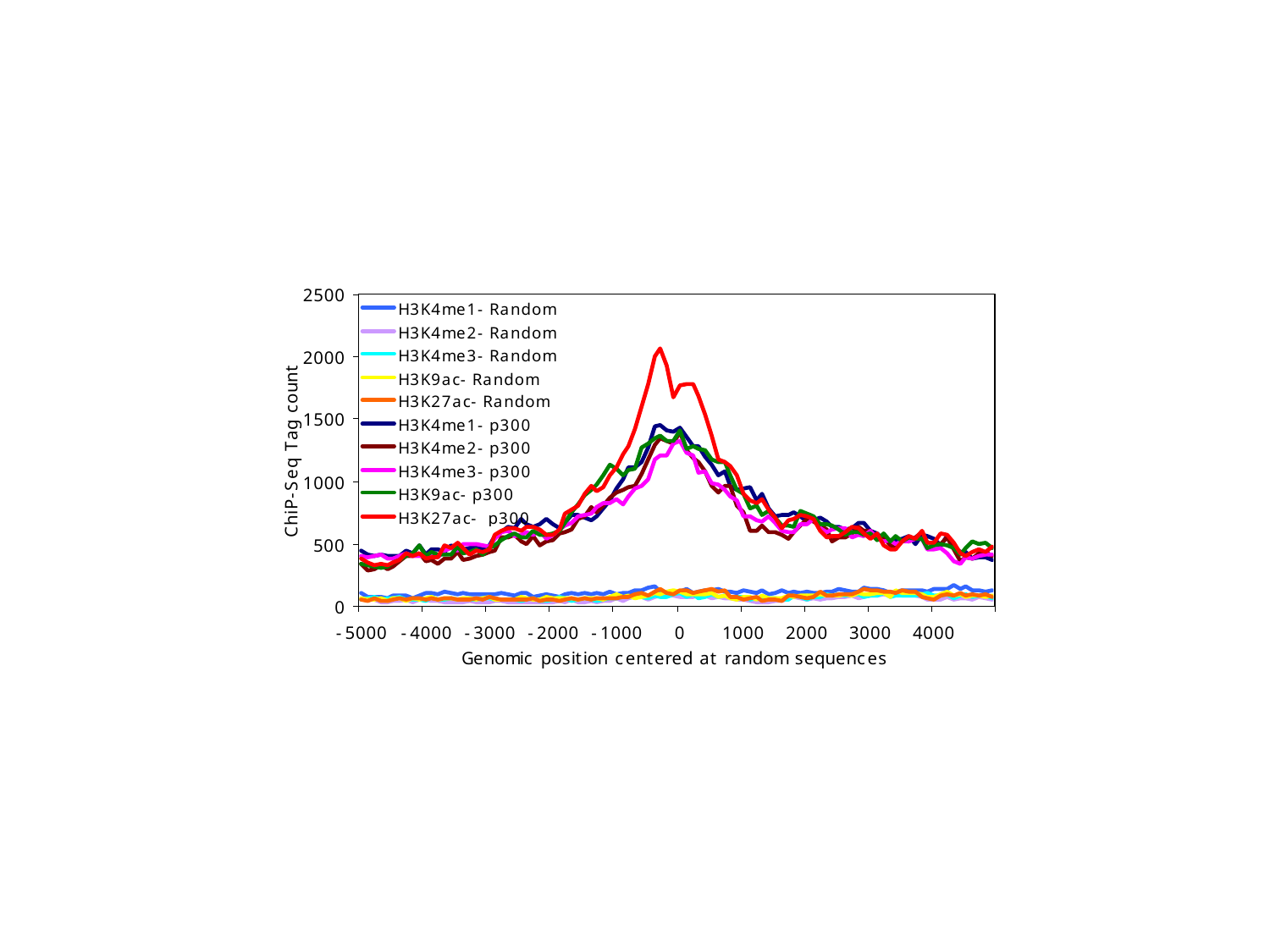

Supplement: Figure S2 — Control 2: Histone modification enrichment patterns at p300 binding sites versus random genomic loci. Epigenetic histone modification levels at 137 p300 binding sites as well as 137 random genomic loci in the K562 cell line are shown. Random genomic loci do not show any discernable pattern of histone modification enrichment compared to p300 binding sites. (PPT) [file pone.0027513.s002.ppt]

## Slide 1
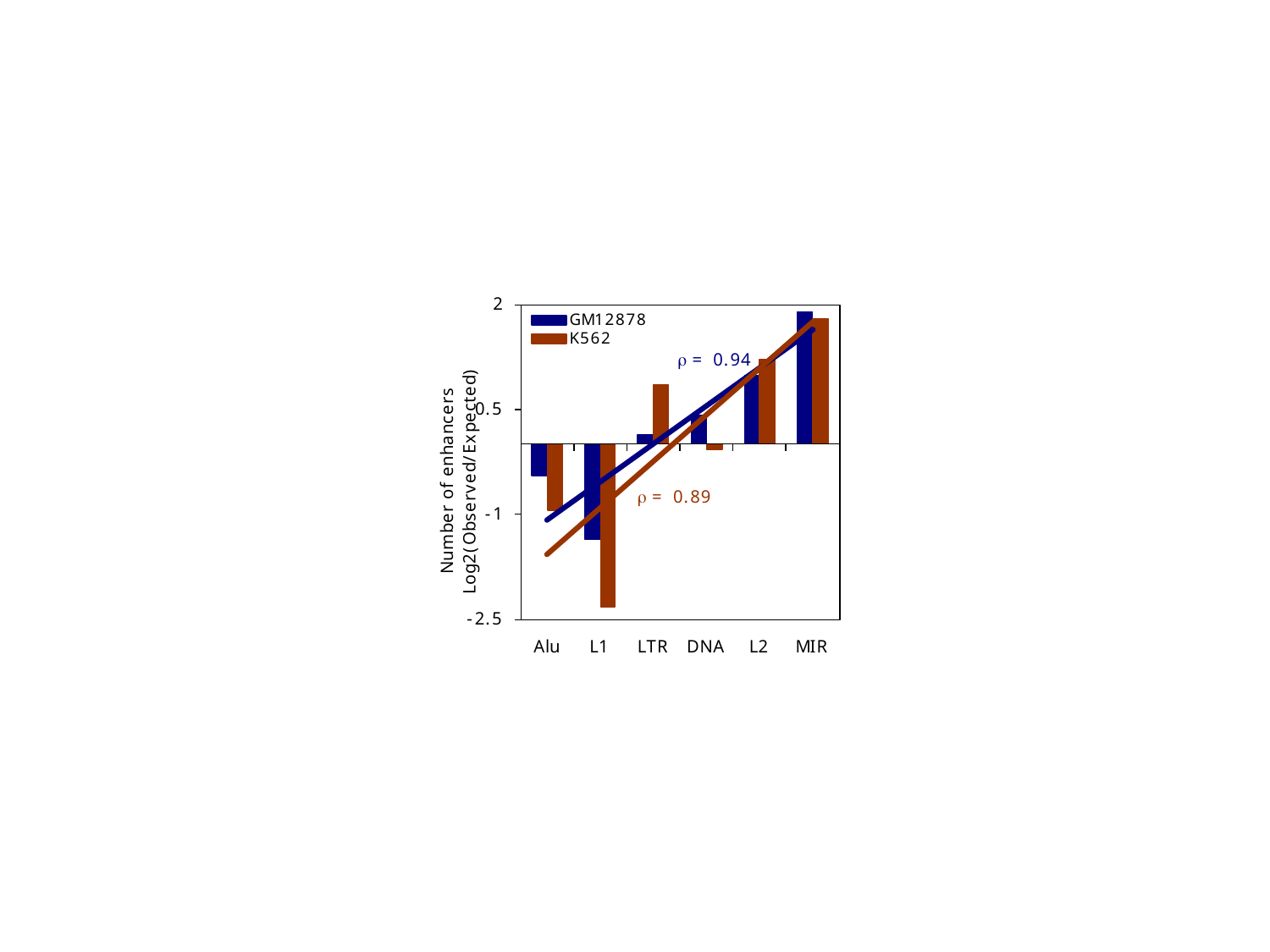

Supplement: Figure S3 — Over and under-represented TE families in contributing enhancers. Number of TE-derived enhancers observed for different TE families in the GM12878 (blue) and K562 (brown) cell lines normalized by the relative genomic abundances of TE families. (PPT) [file pone.0027513.s003.ppt]

## Slide 1
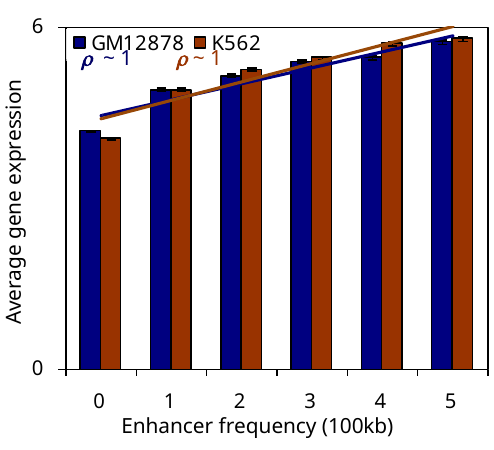

 ~ 1  ~ 1
Average gene expression
Enhancer frequency (100kb)

Supplement: Figure S4 — Functional role of non TE-derived enhancers in regulating gene expression. Average expression levels (y-axis) of genes that are co-located with different numbers of non TE-derived enhancers (x-axis) shown for GM12878 (blue) and K562 (brown) cell lines. (PPT) [file pone.0027513.s004.ppt]

## Slide 1
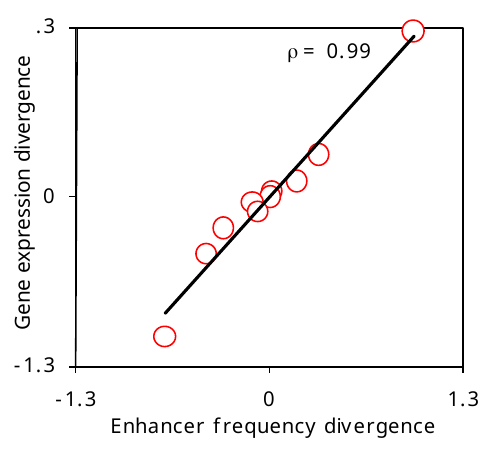

Gene expression divergence

Supplement: Figure S5 — Functional role of non TE-derived enhancers in regulating differential gene expression. Gene expression divergence between GM212878 and K562 (y-axis) is plotted against normalized differenced in the numbers of cell type specific non TE-derived enhancers (x-axis) co-located with the genes. Expression divergence and enhancer frequency divergence between the GM12878 and K562 cell lines is calculated by subtracting the values of K562 from those of GM12878 cell line. (PPT) [file pone.0027513.s005.ppt]

## Slide 1
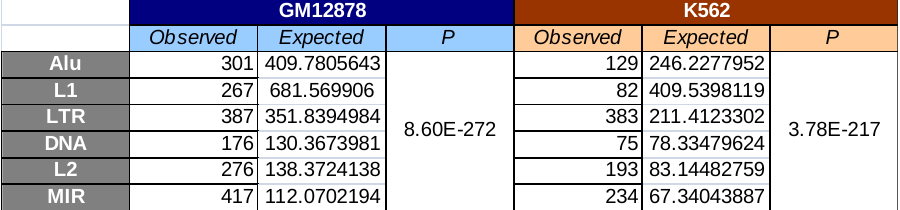

Supplement: Table S4 — χ2 statistics for over and under represented TE families in contributing enhancers. (PPTX) [file pone.0027513.s009.pptx]
